# Serological differentiation between Mpox infection and vaccine-induced immunity: a mini-review

**DOI:** 10.3389/fimmu.2026.1870994

**Published:** 2026-08-12

**Authors:** Zhumagali Koshemetov, Yerbol Bulatov, Orazbek Serikbayov, Kuandyk Zhugunissov

**Affiliations:** Research Institute for Biological Safety Problems, National Holding QazBioPharm, Gvardeyskiy, Kazakhstan

**Keywords:** humoral immune response, mpox, MPXV, serological differentiation, vaccine-induced immunity, viral antigens

## Abstract

In recent years, mpox has emerged as a significant global public health threat. The widespread distribution of the virus, coupled with the legacy of historical smallpox vaccination, has underscored the critical need to serologically distinguish between natural mpox infection and vaccine-induced humoral immune responses. However, the high antigenic similarity among Orthopoxvirus species leads to the production of cross-reactive antibodies, limiting the differentiation capacity of conventional serological assays. This mini-review evaluates current serological approaches aimed at discriminating between mpox infection and vaccine-induced immunity. Specifically, we analyze multiplexed immunoassays, comparative ratios based on orthologous antigens, machine learning-supported serological models, peptide-based platforms, and recombinant protein-based enzyme-linked immunosorbent assay (ELISA) methods. The findings of this review indicate that multiplex immunoassays, orthologous antigen ratio-based approaches, and machine learning algorithms exhibit the highest discriminatory potential for distinguishing natural mpox infection from vaccine-induced immune responses. In addition, MPXV A27L and ATI-N-CPXV antigens may be considered promising candidates for serological differentiation, although their performance requires further investigation.

## Introduction

Mpox is a zoonotic disease caused by monkeypox virus (MPXV), an Orthopoxvirus closely related to variola virus (VARV), vaccinia virus (VACV), and cowpox virus (CPXV) (1–18). Previously considered a sporadic infection limited to Central and West Africa, its geographic spread has expanded over the past two decades, culminating in a global outbreak in 2022–2024 and a World Health Organization-declared Public Health Emergency of International Concern (19–29). MPXV is transmitted from animals to humans and from human to human (1, 30–42). Disease severity depends on age, immune status, and underlying comorbidities. The case fatality rate varies by viral clade and has been reported at 3.9–4.9% in the current epidemic (43–53). The global spread of MPXV has increased the need for reliable diagnostic and surveillance methods.

Molecular diagnostic methods, particularly real-time polymerase chain reaction, are considered the gold standard for diagnosing mpox infection (54, 55), while antigen-based rapid tests are being actively developed and improved (56, 57). Serological methods and Orthopoxvirus antigens are widely used in seroepidemiological monitoring, population immunity assessment, and the study of long-term immunological memory (18, 58–65). Recent studies are focused on distinguishing natural mpox infection from vaccine-induced immune responses.

A major challenge in Orthopoxvirus serology is the high antigenic similarity among MPXV, VACV, VARV, and CPXV (**Table 1**), which leads to pronounced antibody cross-reactivity (66). As a result, conventional serological assays cannot reliably distinguish antibodies induced by mpox infection from those generated after smallpox vaccination. To overcome this limitation, recent studies have proposed multiplex assays, orthologous antigen-ratio approaches, machine learning-based methods, as well as peptide-based and recombinant platforms (93–101, 112, 113).

**Table 1:** Characteristics of major MPXV serological antigens and their orthologs.

Virion form	MPXV antigen	Ortholog		Function	Length	kDa	Identity (%)	References
*IMV*	A29L	A27L	VACV	virus-cell attachment, viral entry, and virus release from cells	110	12	94.54	Sagdat et al. (67)
							93.6	Gao et al. (68)
							94.54	Tan et al. (69)
	E8L	D8L		cell surface attachment and viral entry	304	32	97.74	Sagdat et al. (67)
							94.7	Tan et al. (69)
	M1R	L1R		myristoylated membrane protein involved in viral particle assembly and cellular entry	250	27.3	98.40	Sagdat et al. (67)
							98.8	Gao et al. (68)
							98.4	Tan et al. (69)
	H3L	H3L		host cell attachment, promotion of viral infectivity, and viral morphogenesis.	324	37.5	93.52	Sagdat et al. (67)
							93.5	Gao et al. (68)
	A27L	ATI-N	CPXV	Truncated homolog of the cowpox virus A-type inclusion protein	696	80.8	36.51	Deng et al. (70)
*EEV*	A35R	A33R	VACV	EV membrane disruption, and effective cell-to-cell spread of viral particles	181	20.6	95.03	Sagdat et al. (67)
							94.5	Gao et al. (68)
							95.03	Tan et al. (69)
	B6R	B5R		efficient spreading of infection, regulation of complement system of the host cell, and virus release from the cell.	317	35.1	96.53	Sagdat et al. (67)
							96.2	Gao et al. (68)
							96.53	Tan et al. (69)
*-*	B21R	B22R	VARV	immunomodulatory membrane-associated glycoprotein containing a cadherin-like domain	1879	212.3	84	Dubois et al. (71) Jahankir et al. (72)

IMV, intracellular mature virion; EEV, extracellular enveloped virion.

Therefore, the aim of this mini-review is to critically evaluate current serological strategies for distinguishing mpox infection from vaccine-induced immunity and to assess their discriminatory performance, advantages, and limitations.

## Serological background of orthopoxviruses

Following the discontinuation of universal smallpox vaccination, population-level immunity against Orthopoxviruses has progressively declined due to the reduction in vaccinated individuals and the increasing proportion of non-immunized cohorts (73). This demographic transition has contributed to the wider spread of mpox in recent decades (74, 75). Despite this decline, multiple studies indicate that vaccine-induced immunity from historical smallpox vaccination can persist for decades. For example, Hammarlund et al. reported that more than 90% of vaccinated individuals retained stable humoral and T-cell-mediated immune responses for 25–75 years (76). Other studies have demonstrated persistence of T-cell immunity for up to 50 years (77), while long-term maintenance of humoral immunity has been observed for 13–88 years (78).

Recent data further confirm the presence of cross-reactive immune responses within the Orthopoxvirus genus. Liu et al. reported that 89.6% of individuals vaccinated with historical smallpox vaccination had detectable IgG antibodies against MPXV, while 70.1% exhibited neutralizing activity (79). Other studies have confirmed that smallpox vaccination significantly reduces both the risk of mpox infection and disease severity (80). *In vitro* studies further demonstrate that antibodies induced by historical smallpox vaccination can effectively neutralize MPXV (81–83). Moreover, real-world evidence indicates that the third-generation JYNNEOS (MVA-BN) vaccine provides effective protection against mpox infection along with a favorable safety profile (84).

However, the protective effect of historical vaccination is gradually declining at the population level, mainly due to the increasing proportion of unvaccinated individuals (85). Epidemiological data from the Democratic Republic of the Congo showed that approximately 90% of individuals infected with mpox had no prior vaccination history (86), while another study reported that unvaccinated populations had a 2.73–9.64-fold higher risk of infection compared to vaccinated groups (87). Seroprevalence studies indicate that residual immunity is geographically variable and generally limited, with rates of 15.16% in France, 0.5% in Bolivia, 3.81% in Laos, and 17.25% in Mali (88). In the current epidemiological context of mpox, children represent a particularly vulnerable population, as high infection rates have been reported among individuals aged 10–15 years in regions such as Zaire and Burundi (30% and 47%, respectively) (85, 89).

In summary, vaccine-induced immunity against Orthopoxviruses persists for decades and provides protection against mpox; however, its gradual population-level decline, driven by reduced vaccination coverage and more non-immunized individuals, has become a key factor in the re-emergence and spread of mpox.

## Multiplex immunoassays

The global spread of mpox has increased the need for serological methods to distinguish natural infection from vaccine-induced immunity (serological differentiation). High antigenic similarity among Orthopoxviruses limits single-antigen assays (90, 91), while multiplex assays improve accuracy by assessing responses to multiple antigens (92).

In Hicks et al., MPXV (A35R, B6R, E8L, H3L, M1R) and VACV (A33R, B5R, D8L) antigens showed high sensitivity but strong cross-reactivity, limiting differentiation of infection from vaccine-induced immunity. In contrast, MPXV A29L showed better discrimination with 88.9% sensitivity and 90% specificity (93). Likewise, Jones et al., a multiplex immunoassay incorporating eight viral antigens demonstrated high sensitivity for detecting Orthopoxvirus seropositivity, while the MPXV A27L antigen showed strong discriminatory performance in distinguishing naturally infected individuals from vaccinated cohorts (sensitivity 87.5%, specificity 96.84%). The authors explained this by the full replication of MPXV during natural infection, leading to complete expression of the A27L antigen. In contrast, the Modified Vaccinia Ankara-Bavarian Nordic (MVA-BN) vaccine is based on a non-replicating platform, resulting in a limited immune response against the A27L antigen (94). Consistent findings were also reported in a subsequent study by Jones et al., where the MPXV A27L antigen achieved a sensitivity of 88% and a specificity of 97%. These results further confirm its value as a reliable marker for serological differentiation (95).

Subsequent studies have shown that analyzing antibody response ratios to orthologous MPXV and VACV antigens provides a more reliable approach for serological differentiation (96–98). Reed et al. identified VACV (D8L, B5R) MPXV B6R as effective markers for detecting Orthopoxvirus exposure; however, substantial cross-reactivity limited their ability to distinguish infection- and vaccine-induced immune responses. In contrast, the antibody response ratio of MPXV A35R/VACV A33R significantly improved discriminatory performance, achieving a sensitivity of 97% and a specificity of 96% (96). Comparable findings have been reported in other studies, where models based on the ratios of antibody responses to paired MPXV (A35R, B6R, E8L) and VACV (A33R, B5R, D8L) orthologous antigens achieved an overall accuracy of 88.9%, a sensitivity of 90.48%, and a specificity of 96.72% for distinguishing different exposure groups (97). Byrne et al. demonstrated that combining the antibody response ratio of MPXV B6R and VACV B5R with the MPXV A27L antigenic response enables differentiation between natural infection and vaccination (sensitivity 89%, specificity 80%) (98). Yates et al. reported that a platform integrating MPXV-specific peptides with orthologous antigens achieved high diagnostic performance, with a sensitivity of 92% and a specificity of 98%. However, its discriminatory performance declined in individuals living with human immunodeficiency virus (HIV), with an overall accuracy of 83% (99).

Overall, multiplex immunoassays provide strong discriminatory capability for differentiating mpox infection from vaccine-induced immunity. However, variability in antigen panels, study designs, and analytical approaches currently prevents the identification of a single universally applicable method.

## Machine learning-supported serological multiplex assays

In recent years, the integration of multiplex serological assays with machine learning has been used for serological differentiation, enabling the detection of latent patterns in immunological datasets.

In this context, Surtees et al. demonstrated that an eight-antigen (ATI-N-CPXV, ATI-C-CPXV, D8L, E8L, A33R, A35R, VACV lysate, HEp-2 lysates, comprising Delta-VACV) serological panel distinguished immune response origins with high accuracy (sensitivity 92%, specificity 88%). Notably, the ATI-N-CPXV antigen was identified as the principal marker for serological differentiation. A pronounced antibody response to this antigen was observed exclusively in individuals with a history of mpox infection (100). Comparable findings were obtained in a subsequent study using a 15-antigen multiplex serological panel (A27L/A29L, L1R/M1R, D8L/E8L, H3L, A33R/A35R, B5R/B6R, A5L, ATI-C/-N, Delta), which achieved high accuracy for serological differentiation (Area Under the Curve (AUC) = 0.89). Consistent with the previous study, a robust antibody response to ATI-N was detected only in infected cohorts (101).

The ATI antigen is considered an immunodominant protein of CPXV and is highly conserved across more than 90% of Orthopoxvirus species (102). It has also been described as a homolog of the MPXV A27L antigen (100). ATI-N is highly expressed during viral replication and exhibits strong immunogenic properties. During natural mpox infection, complete viral replication allows ATI-N-derived epitopes to be broadly presented to the host immune system, resulting in the production of ATI-N-specific antibodies. In contrast, non-replicating MVA-BN-based vaccines do not undergo a complete replication cycle and therefore fail to induce a measurable immune response against the ATI antigen. Consequently, the ATI-N antigen represents a valuable serological marker for distinguishing infection-induced immunity from vaccine-induced immunity. Furthermore, it has also been successfully used to differentiate immune responses induced by the Dryvax and MVA-BN vaccines (103).

In both studies, the Gradient Boosting Classifier (GBC) has shown consistently high performance in serological classification tasks. This algorithm is widely used for multidimensional biomedical data analysis and supports accurate differentiation between immune response types.

To facilitate comparison of the serological approaches discussed above, their principal characteristics, target antigens, diagnostic performance, advantages, and limitations are summarized in **Table 2**.

**Table 2:** Comparative characteristics of serological approaches for differentiating natural mpox infection from vaccine-induced immunity.

Assay platform	Key discriminatory marker	Sensitivity (%)	Specificity (%)	Advantages	Limitations	Validation type	Sample size (n)	Cohort composition	Vaccine platform	References
MSD V-PLEX Orthopoxvirus Panel 1 Electrochemiluminescence immunoassay (ECLIA)	MPXV A29L	88.9	90.0	Provides effective serological differentiation.	Discriminatory performance depends primarily on the MPXV A29L antigen	Internal validation	328	MP (39); MVA (74: PD1, 9; PD2, 65); PC (215)	MVA-BN (IMVANEX)	Hicks et al. (93)
Luminex MagPlex^®^	MPXV A27L	87.5	96.84		Discriminatory performance depends primarily on the MPXV A27L antigen	Internal validation	534	MP (24); MVA (75: PD1, 7; PD2, 68); Pre-MVA (18); PC (224); CMV (52); EBV (34); VZV (44); RF (63)	MVA-BN (IMVANEX)	Jones et al. (94)
Luminex MagPlex^®^	MPXV A27L	88	97			Internal validation	534	MP (24); MVA (75: PD1, 7; PD2, 68); Pre-MVA (18); PC (224); CMV (52); EBV (34); VZV (44); RF (63)	MVA-BN (IMVANEX)	Jones et al. (95)
MSD V-PLEX Orthopoxvirus Panel 1	MPXV A35R/VACV A33R	97	96	Enables serological differentiation using the antibody response ratio of a single orthologous antigen pair	Individual antigens exhibit limited discriminatory performance	Internal validation	257	MP (26); MVA (52); PC (68); Ru (111)	MVA-BN (JYNNEOS)	Reed et al. (96)
MSD V-PLEX Orthopoxvirus Panel 1	MPXV A35R/VACV A33R MPXV B6R/VACV B5R MPXV E8L/VACV D8L	90.48	96.72	Improves discrimination by combining antibody response ratios from three orthologous antigen pairs	Different cut-off values are required for different antigen pairs	Internal validation	136	MP (22); MVA (53: PD1, 42; PD2, 11); HC (61)	MVA-BN (JYNNEOS)	Pettke et al. (97)
MSD ECLIA	MPXV B6R/VACV B5R+ MPXV A27LL	89	80	Combines orthologous antigen ratios with the MPXV A27LL marker to improve discriminatory performance	Relatively low specificity	Internal validation	295	MP (28); MVA (167); HC (78); ChV (22)	MVA-BN (JYNNEOS)	Byrne et al. (98)
Luminex Microsphere Immunoassay	MPXV B21/22R, A33R/A35R, B5R, L1R, H3L	93	98	Selective targeting of MPXV-specific immunodominant epitopes provides high sensitivity while minimizing cross-reactivity	Reduced sensitivity in HIV-positive individuals	Internal validation	396	MP (40); MVA (13); ACA (2); HC (199); HIVP (131); HIVN (12)	MVA-BN (JYNNEOS)/ACAM2000	Yates et al. (99)
Luminex xMAP^®^	ATI-N-CPXV	92	88	Identifies complex immune response patterns, enabling accurate discrimination of natural mpox infection	Requires dedicated machine learning algorithms and additional computational analysis	Internal validation with independent validation cohort	537 (acute cohort); 859 (epidemiological cohort) independent validation cohort (143)	Acute cohort: MP (307); MVA vaccinated (54); HC (176). Epidemiological cohort: MP (59); MVA (476); HC (324) Independent validation cohort: MP (55); MVA (35); pre-MVA (3); HC (50).	MVA-BN	Surtees et al. (100)
Luminex xMAP^®^	ATI-N-CPXV	AUC of 0.89				Internal validation	531 (acute cohort); 859 (epidemiological cohort)	Acute cohort: MP (307); MVA (48); HC (176) Epidemiological cohort: MP (59); MVA (476); HC (324)	MVA-BN	Stern et al. (101)
Peptide-Based ELISA	(A56) A56-312/313 (A33R) A33-119/129 (E3) E3-62/80	86	90	Selective targeting of MPXV-specific immunodominant epitopes	Limited number of vaccinated cohorts evaluated	Internal validation	NR	MP (35); vaccinated (26); HC (95) VARV archival sera (NR)	Specific vaccine not reported for the human cohort.	Taha et al. (112)
Recombinant protein-based ELISA	MPXV A27L	91.3	98.86	Simple assay design with high specificity	Cross-reactivity was observed with replication-competent vaccinia-based vaccines	Internal validation	766	MP (46); MVA (60); ACA (5); PC (256); CMV (99); EBV (100); VZV (100); RF (100)	MVA-BN/ ACAM2000	Otter et al. (113)

MP, Mpox-infected; MVA, MVA-BN-vaccinated; PD1, Post-dose 1; PD2, Post-dose 2; ACA, ACAM2000-vaccinated; HC, healthy controls; PC, paediatric control; Pre-MVA, Pre-MVA-BN vaccination; ChV, Childhood vaccine; VZV, Varicella-zoster virus-positive; CMV, Cytomegalovirus-positive; EBV, Epstein–Barr virus-positive; RF, Rheumatoid factor-positive; HIVP, HIV antibody-positive; HIVN, HIV antibody-negative; Ru, Rubella IgG positive; NR, Not Reported.

## Discussion

Serological differentiation between mpox infection and vaccine-induced immunity remains challenging due to the extensive antigenic conservation among Orthopoxviruses, which results in substantial antibody cross-reactivity and consequently reduces the specificity of conventional serological assays.

Multiplex immunoassays provide an effective approach to overcome this limitation. Early studies showed that individual antigens possess measurable discriminatory capacity (93–95); however, subsequent studies demonstrated that extensive Orthopoxvirus cross-reactivity limits single-antigen approaches. Consequently, orthologous antigen ratios have emerged as a more reliable strategy for serological differentiation (96–98).

MPXV A29L antigen has been described as an effective marker for detecting immune responses associated with natural infection (93). The diagnostic performance of the A29L antigen in recombinant ELISA systems was limited. Although the Abbexa and ProteoGenix assays showed high sensitivity (88% and 100%, respectively), both exhibited 0% specificity, indicating a high false-positive rate. In addition, MSD multiplex analysis demonstrated low discriminatory performance for A29L (76% sensitivity, 64% specificity), highlighting its limited reliability for serological differentiation (104). In some studies, it did not elicit an immune response in non-replicating vaccine platforms; however, it induced a strong immunogenic response in live vaccine platforms (ACAM2000, VACV-Tiantan) (105, 113). In contrast, the MPXV A27L antigen has demonstrated high discriminatory performance in multiplex assay panels (94). Overall, the available evidence suggests that the MPXV A29L antigen has limited potential as a discriminatory serological marker, whereas the MPXV A27L antigen may be considered a promising serological marker.

The use of individual antigens is insufficient for the reliable differentiation of immune responses; therefore, subsequent studies have proposed approaches based on ratios of orthologous antigens. However, this method has several limitations, as a separate cut-off value must be defined for each antigen ratio, and data interpretation requires additional calculations. For example, the cut-off value for MPXV A35R/VACV A33R was 1.6 (96), whereas the corresponding values for MPXV A35R/VACV A33R, MPXV B6R/VACV B5R, and MPXV E8L/VACV D8L were 1.50, 1.05, and 1.10, respectively (97). In addition, a cut-off value of 0.92 was defined for the MPXV B6R/VACV B5R and MPXV A27L combination (98). Furthermore, the discriminatory capacity of the orthologous-ratio approach may be reduced in individuals with a history of both vaccination and subsequent infection. Evolutionary changes in MPXV surface proteins during future outbreaks may alter antibody responses against MPXV and VACV orthologs, potentially affecting the robustness of this approach (96). The performance of this method may depend on the study population and experimental conditions, as differences in background antibody reactivity among populations require re-standardization of cut-off values (97). In addition, the faster decline of antibody levels after vaccination compared with infection-induced immunity highlights the importance of considering the time factor when differentiating immune responses using this approach (98). Thus, inconsistent cut-off values, the challenges identified by the authors, and insufficient methodological standardization limit the reproducibility of the results.

Yates et al. identified the B21R/B22R peptides as key discriminatory markers for mpox infection; although conserved across several Orthopoxvirus species, they are absent from VACV (71, 99). Therefore, interpretation of assay results based on this target may be challenging in regions where Orthopoxviruses are endemic (106). In addition, deletions in the B21R gene were reported during the 2022 mpox outbreak (107). The presence of such genetic variations in circulating strains may reduce the sensitivity of assays targeting the B21R peptide.

Despite the limitations mentioned above, multiplex immunoassay approaches significantly improve discriminatory accuracy by enabling the simultaneous assessment of antibody responses against multiple antigens. This approach has been validated in several infectious diseases. In COVID-19, multiplex panels targeting the spike protein, its Receptor-Binding Domain, and nucleocapsid antigens accurately differentiate infection-induced and vaccine-induced immune responses (108). In HIV, multiplex profiling enables comprehensive immune characterization and improves disease staging (109). In Hepatitis C, multi-antigen assays enhance early diagnosis compared to single-marker tests (110), while in Influenza they effectively differentiate infection-derived and vaccine-induced immune responses (111).

Machine learning approaches enhance serological classification accuracy by enabling the analysis of high-dimensional immunological data. The GBC algorithm identifies latent patterns and effectively discriminates between immune responses induced by natural infection and vaccination; in this context, the ATI-N-CPXV antigen has been identified as a key marker. However, these approaches are limited by inter-study variability and dependence on high computational resources (101, 102).

Taha Y. et al. used a peptide-based ELISA incorporating three peptides (A56, A33, and E3) for serological differentiation, achieving a sensitivity of 86% and a specificity of 90%. However, the study included a limited vaccinated cohort (n=26), which is insufficient to fully capture immunological variability (112). Interestingly, Otter et al. reported that a recombinant ELISA targeting the MPXV A27L antigen demonstrated high discriminatory performance in individuals vaccinated with MVA-BN (sensitivity 91.3% and specificity 98.86%). However, in recipients of the live vaccinia virus vaccine ACAM2000, partial antibody reactivity to this antigen was also observed, indicating limited discrimination in this vaccination platform (113).

## Interpretation of key markers for serological differentiation

MPXV A29L is a major surface protein of the mature virion and elicits a robust antibody response following natural mpox infection. Because the VACV A27L protein encoded by the replication-competent ACAM2000 vaccine is an ortholog of MPXV A29L, antibodies induced by vaccination exhibit cross-reactivity with the MPXV A29L antigen (113–115). In addition, antibodies against MPXV A29L have been detected following both one- and two-dose MVA-BN vaccination regimens (96, 97, 116). Taken together, because antibodies to MPXV A29L are induced by both natural infection and vaccination with ACAM2000 or MVA-BN, this antigen has limited diagnostic value as a stand-alone serological marker for distinguishing natural mpox infection from vaccine-induced immunity.

MPXV B21R is an ortholog of the VARV B22R protein and is absent from VACV (76, 117). Consequently, B21R/B22R-specific antibodies are not expected to be induced following vaccination with either ACAM2000 or MVA-BN. Several studies have identified B21R/B22R peptides as promising serological markers for distinguishing natural mpox infection from vaccine-induced immunity (71, 118). However, a 6,881-bp deletion affecting the MPXV B21R gene has been reported in the WA-UW-074978 isolate (107), while another MPXV isolate contained an approximately 15-kb deletion resulting in the loss of multiple B21R open reading frame (ORF) regions (119). In addition, a strain with deletions spanning MPXVgp184-MPXVgp187 has been described, although these changes did not disrupt the ORF structure (120). Although Yates et al. demonstrated the high discriminatory performance of B21R/B22R peptides, the number of vaccinated samples included in their study was limited (n = 15) (99). Nevertheless, the diagnostic reliability of these markers requires further validation in large independent cohorts representing different vaccine platforms and post-vaccination time intervals.

MPXV A27L is homologous to the N-terminal region of the CPXV ATI-N protein and has no direct ortholog in VACV (66). ATI-N, which is expressed in MPXV but not in MVA-BN, represents an infection-associated antigen (121). Furthermore, antibodies against MPXV A27L have not been detected even after three doses of the MVA-BN vaccine (113). Therefore, MPXV A27L and ATI-N represent promising serological markers for distinguishing natural mpox infection from vaccine-induced immunity.

## Limitations of current evidence

Several limitations should be considered when interpreting the literature on serological differentiation. First, there is no internationally accepted reference antigen, assay, or diagnostic cut-off. Consequently, direct comparison of findings across studies remains difficult.

Secondly, cohorts across studies differ substantially in size, demographic composition, and origin (geographic region, vaccination status, and time since infection), which limits direct comparability of results. MPXV cohorts are heterogeneous and derived from diverse populations (93–101, 112, 113). Control groups include pediatric samples, previously vaccinated individuals, seropositive subjects with other viral infections (Cytomegalovirus, Epstein-Barr virus, Varicella-zoster virus), and HIV-positive populations (93–96, 99). Vaccine cohorts mainly involve MVA-BN and ACAM2000, while in MVA-BN studies, samples were collected at different time points, complicating direct comparison of immune responses. In addition, some studies used repeated samples from the same participants across multiple time points, leading to discrepancies between participant numbers and analyzed samples (95, 100, 101, 113). Overall, substantial cohort heterogeneity reduces comparability across studies.

Third, in studies based on orthologous antigen ratios, cut-off values are not uniform, which complicates the comparison of results.

In addition, although peptide-based ELISA assays have demonstrated high discriminatory performance, the limited size of vaccinated cohorts prevents a comprehensive assessment of the assay’s reliability (112). Therefore, further studies in diverse populations and larger cohorts are required to evaluate its performance.

Finally, most studies reported sensitivity and specificity values based on internal validation (93–101, 112, 113), while only one study additionally evaluated assay performance using an independent validation cohort (100). Therefore, findings based primarily on internal validation cannot fully confirm the reliability of these methods in real-world clinical settings, highlighting the need for further independent external validation.

## Conclusion and future directions

In conclusion, it is difficult to identify a universal serological method or marker that reliably distinguishes natural mpox infection from vaccine-induced immunity. Current evidence indicates that multiplex immunoassays, orthologous antigen ratio-based approaches, and machine learning models provide the highest discriminatory performance. However, their performance depends on the selected antigens, assay platforms, and characteristics of the study cohorts; therefore, these approaches still require further standardization and independent validation. In addition, current scientific evidence supports considering MPXV A27L and ATI-N-CPXV antigens as promising candidates for serological differentiation, although their discriminatory potential requires confirmation through independent external validation studies.

Future research should focus on establishing an internationally accepted reference antigen panel and unified diagnostic cut-off values.
